# Supplementary material for: Identifying A- and P-site locations on ribosome-protected mRNA fragments using Integer Programming
Source: Sci Rep. 2019 Apr 18;9:6256. doi: 10.1038/s41598-019-42348-x (PMC6472398; doi:10.1038/s41598-019-42348-x)
Supplement: Supplementary file 1 — Supplementary Information [file 41598_2019_42348_MOESM1_ESM.pdf]

Supplementary Information - Identifying A- and P-site locations on ribosome-protected mRNA fragments using Integer Programming

Nabeel Ahmed[1, ¶], Pietro Sormanni[2, ¶], Prajwal Ciryam[2, #], Michele Vendruscolo[2], Christopher M. Dobson[2] and Edward P. O'Brien[1, 3, 4, *]

[1]Bioinformatics and Genomics Graduate Program, The Huck Institutes of the Life Sciences, Pennsylvania State University, University Park, PA, USA

[2]Department of Chemistry, University of Cambridge, Cambridge, UK

[3]Institute for Cyber Science, Pennsylvania State University, University Park, PA, USA

[4]Department of Chemistry, Pennsylvania State University, University Park, PA, USA

¶ These authors contributed equally to this work.

# Present address: Department of Neurology, Columbia University College of Physicians and Surgeons, New York, NY, USA

*To whom correspondence should be addressed. Tel: (814) 867-5100; Fax: (814) 865-2927; Email: epo2@psu.edu

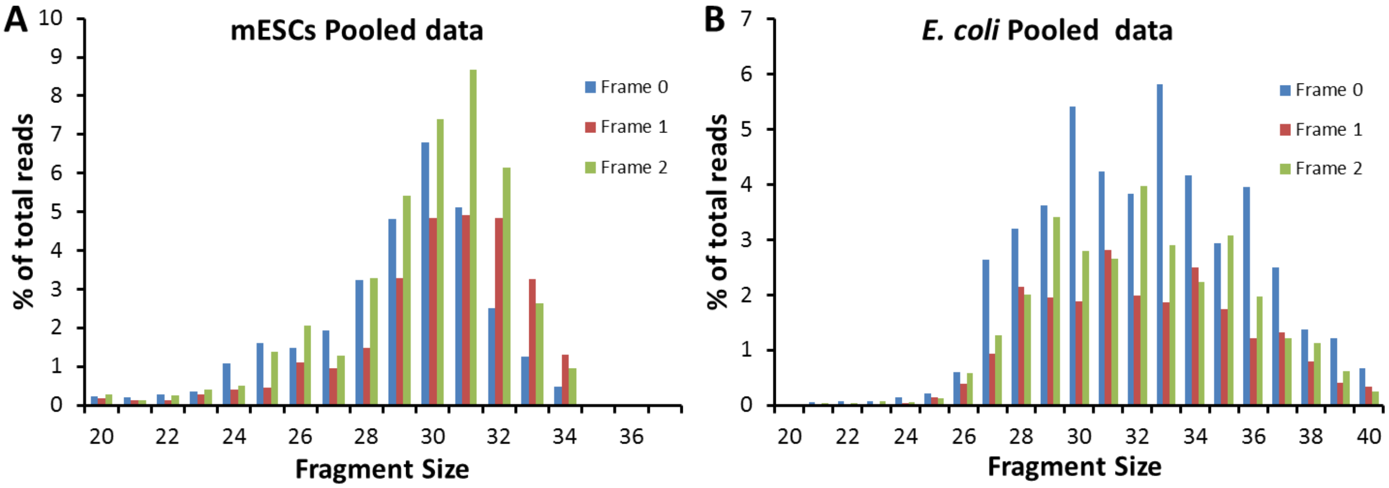

Supplementary Figure S1: Fragment size distribution in **(A)** Pooled Ribo-Seq data in mouse embryonic stem cells (mESCs) and **(B)** Pooled Ribo-Seq data in *Escherichia coli.*

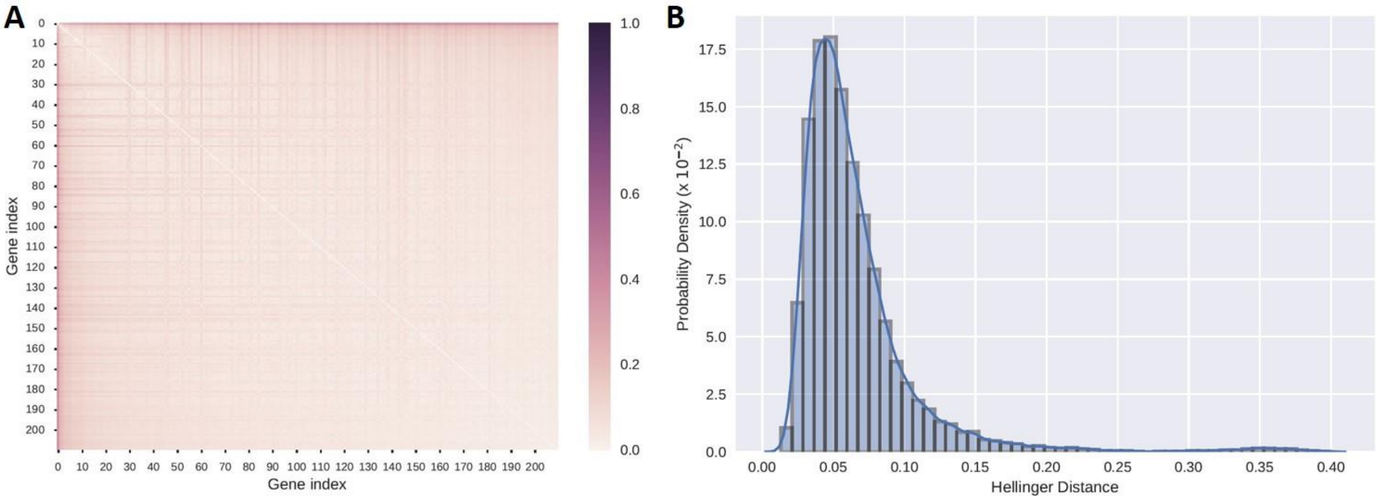

Supplementary Figure S2: Pairwise comparison of fragment-size and frame distributions between genes in *S. cerevisiae*. (A) The heat map reports the pairwise Hellinger distance[1] between the probability densities of the fragment-size and frame distributions of individual genes. Only genes in the Pooled data set that have at least 1 read per codon for fragment sizes between 24 and 34 nt were analyzed, resulting in 210 genes in this analysis. **(B)** The probability density distribution of Hellinger distances reported in (A). The Hellinger distance metric is bound between (0, 1); 0 indicates identical distributions; while 1 indicates the distributions are divergent. All pairwise Hellinger distances are less than 0.45 and only 11% of pairwise distances are greater than 0.1. Hence, the distribution of reads of different fragment sizes and frames are highly similar and therefore exhibit very little dependence on gene identity.

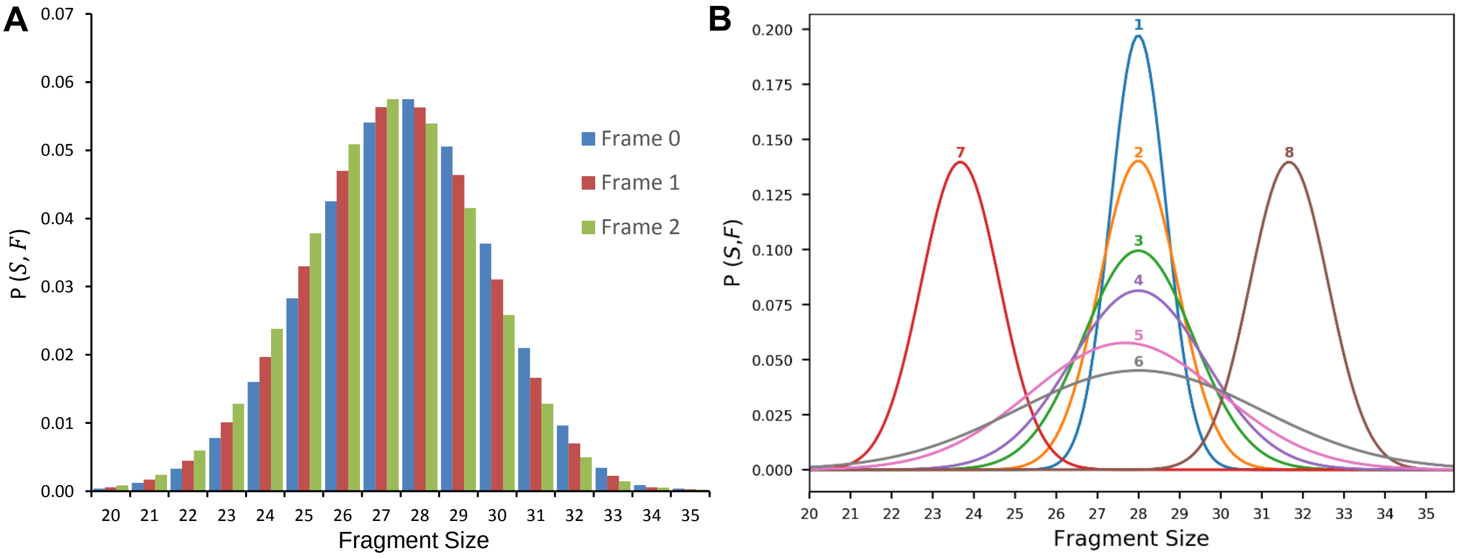

C

Read length Distribution	Input offset table			
	Constant offset of 15	Constant offset of 18	Mixed offsets of 12 and 18	Top offsets from experimental data
1	100%	93%	100%	93%
2	95%	95%	100%	95%
3	96.5%	100%	96.5%	100%
4	100%	100%	100%	97%
5	100%	96%	100%	98%
6	100%	100%	100%	100%
7	100%	100%	100%	100%
8	95%	95%	95%	100%

Supplementary Figure S3: Integer Programming algorithm correctly reproduces the true A-site offsets from Artificial Ribo-Seq data. (A) An example of a Poisson distribution with mode at $(S, F) = (28, 0)$ and a variance $\lambda = 48$ that was used to generate artificial ribosome-protected fragments (see Methods for details). The reads generated from this distribution are subjected to the Integer Programming algorithm for four different input offset tables. These input offset tables are shown in Supplementary Table S4. **(B)** Six read length distributions with their mode at $(28, 0)$ were generated with Poisson variances $\lambda = 4, 8, 16, 24, 48, 80$ and labeled 1 through 6, respectively. The distribution in (A) is the distribution labeled 5 in (B). Two more distributions were generated with variance $\lambda = 8$ but with modes at $(24, 0)$ and $(32, 0)$ and labelled as 7 and 8, respectively. **(C)** The percentage of offsets that the Integer Programming correctly identifies in the artificial Ribo-Seq data created based on the eight read length distributions shown in (B) and for each of the four different input offset tables (see Methods for details) used to generate the artificial Ribo-Seq reads. The four input offset tables and the corresponding output offset tables generated by the Integer Programming algorithm for distribution 5 is shown in Supplementary Table S4.

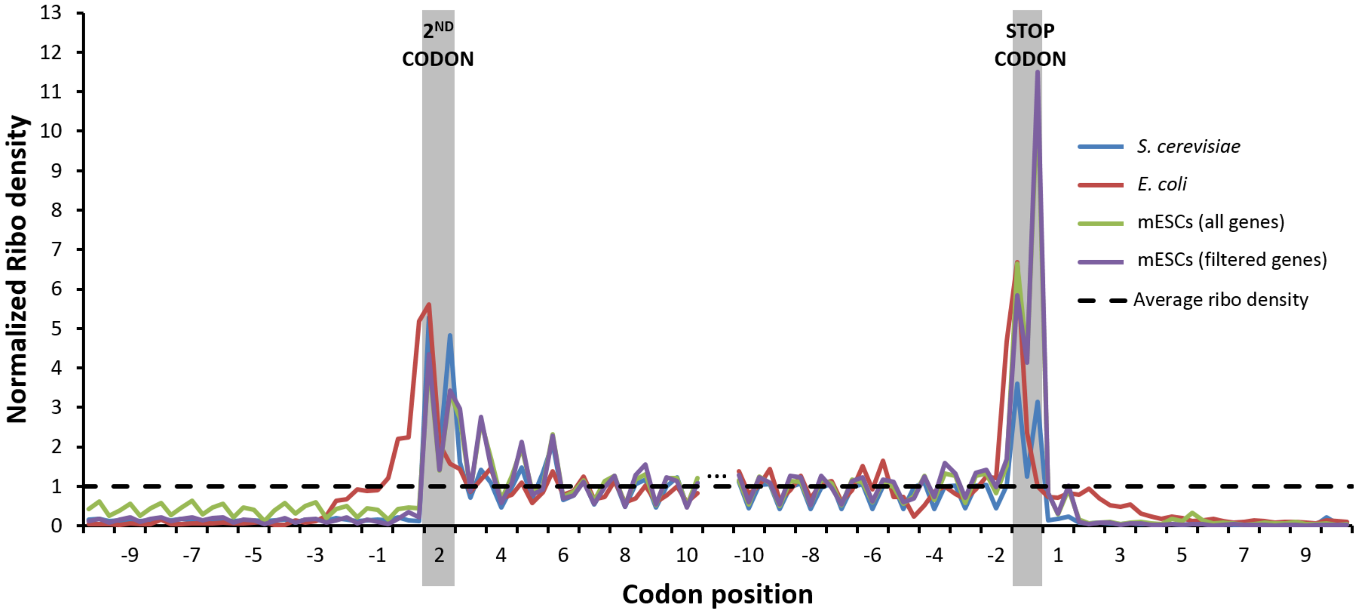

Supplementary Figure S4: Meta-gene analysis in Pooled Ribo-Seq data reveal excess ribosome density in *E.coli* genes beyond CDS regions. A-site profiles are obtained for *S. cerevisiae* using unique offsets from Table 1 obtained after application of Integer Programming algorithm. For *E. coli*, we use a constant offset of 12 nt from 3′ end as used by Woolstenhulme and co-workers[2]. For mESCs, we use the unique offsets from Supplementary Table S6. We plot the meta-gene profiles for all genes dataset as well as for the subset of filtered genes containing only single isoforms with one translation start site.

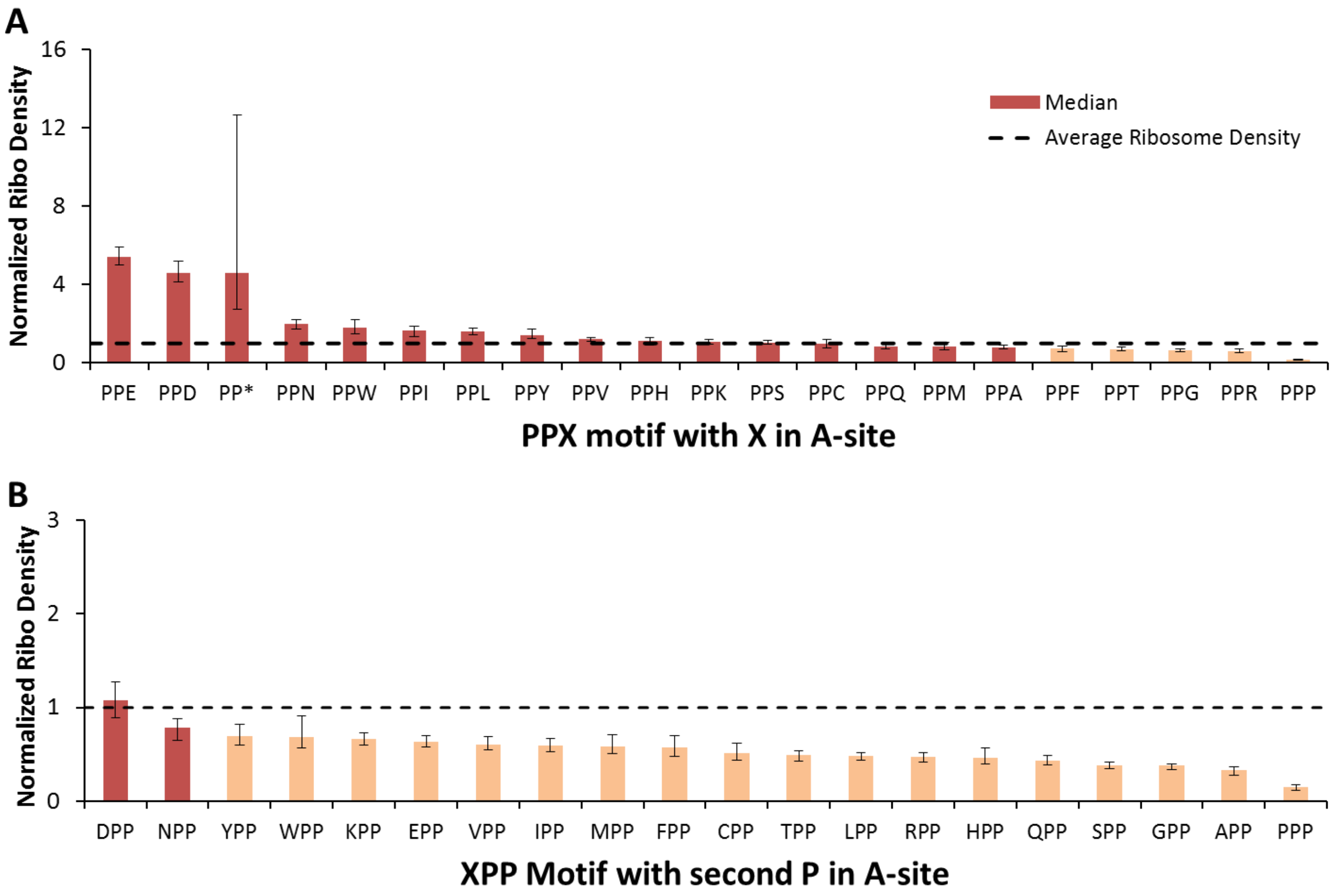

Supplementary Figure S5: Stalling at PPE and PPD motifs are reproduced in mESCs. The median normalized ribosome density is obtained for all instances of **(A)** PPX and **(B)** XPP motifs in which X corresponds to any one of the 20 naturally occurring amino acids (and stop codon for instance PP*). Using a permutation test, we determine if the median ribosome density is statistically different from the average ribosome density. Statistically significant motifs are highlighted in dark red. This analysis was carried out on the Pooled dataset for transcripts in which at least 50% of codon positions have reads mapped to them. Error bars are 95% Confidence Intervals for the median obtained using Bootstrapping[3].

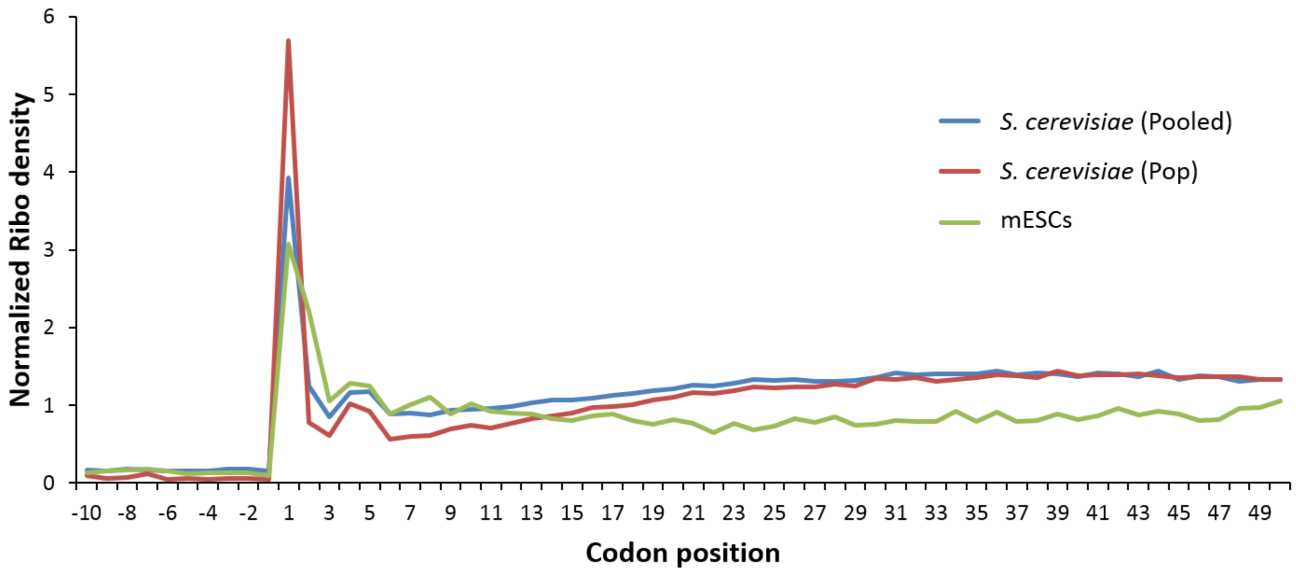

Supplementary Figure S6: Sequence-independent translational pause observed post-initiation in *S. cerevisiae* and mESCs. Meta-gene analysis at the codon level with reads mapped to the P-site are shown. There is a mild but distinct pausing of translation when the 4th and 5th codons are in the P-site. This effect is seen in both Pooled and Pop datasets of *S. cerevisiae* as well as the filtered genes dataset of mouse embryonic stem cells (mESCs).

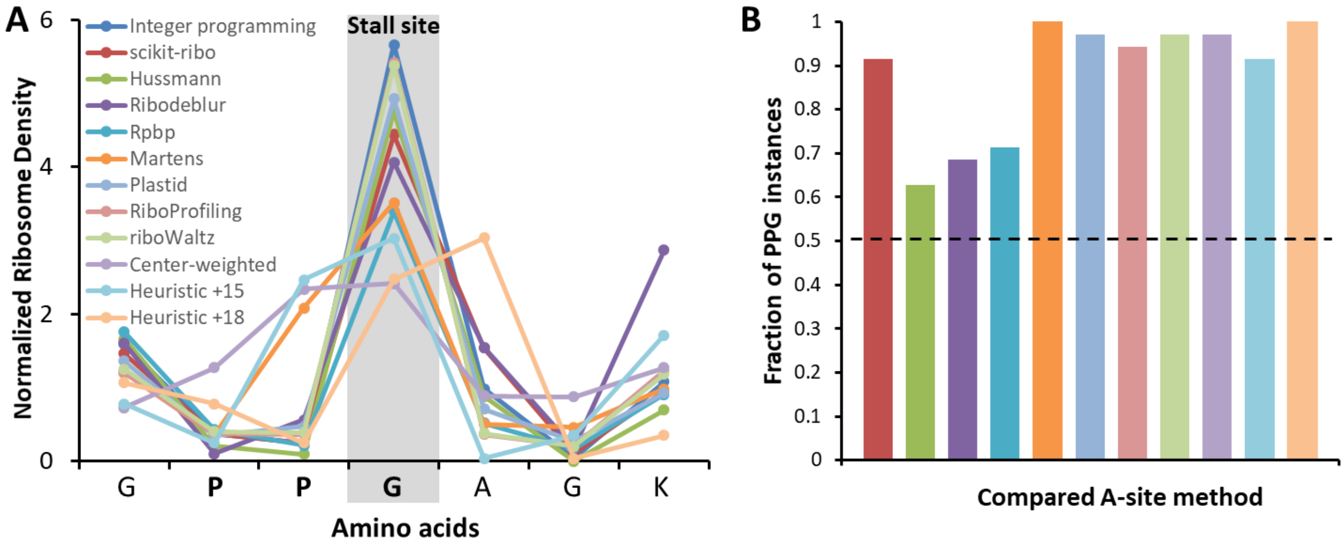

Supplementary Figure S7: The Integer Programming algorithm correctly assigns greater ribosome density to the Glycine residue in PPG motifs than other methods in *S. cerevisiae*. **(A)** Normalized ribosome density at the predicted A-site using different methods to determine A-site is shown for an instance of the PPG motif in gene YDR226W with the Glycine of the PPG motif is at codon position 16, for Pop dataset in *S. cerevisiae*. **(B)** The fraction of PPG instances ($n = 35$) at which Integer Programming method yields greater ribosome density at glycine against the compared method. The color-coding is same as shown in the legend of panel (A). Our method does better if it assigns greater ribosome density in more than half the instances (horizontal line in panel B). Integer Programming yields significantly higher ribosome density at G in the PPG motifs than all other methods (For Hussmann $P = 0.026$, for ribodeblur $P = 0.01$ and for others $P < 10^{-5}$). Two-sided *P*-values were calculated using the Wilcoxon signed rank test. Error bars are 95% Confidence Interval about the median calculated using Bootstrapping[3].

Supplementary Table S1: Number of genes for the various fragment size and frame combinations that meet the criteria of at least 1 read per codon on average in the Pop and Pooled datasets of *S. cerevisiae*.

Fragment size	Pop dataset				Pooled dataset		
	Frame 0	Frame 1	Frame 2		Frame 0	Frame 1	Frame 2
**20**	34	7	57		156	50	98
**21**	45	60	41		224	129	139
**22**	99	55	48		234	115	199
**23**	73	47	95		162	107	290
**24**	58	91	72		161	352	193
**25**	155	69	55		647	251	194
**26**	105	64	175		481	241	916
**27**	159	255	161		1096	2213	878
**28**	1081	248	333		4487	1861	1468
**29**	850	330	1437		3919	1504	4474
**30**	528	876	1139		3184	3041	3835
**31**	276	610	643		2089	2251	3164
**32**	70	279	181		799	1897	1789
**33**	39	82	9		474	1076	322
**34**	1	2	0		237	194	71
**35**	0	0	0		58	40	33

Supplementary Table S2: Initial offset tables after application of Integer Programming algorithm to Pop and Pooled datasets in *S. cerevisiae.*

Fragment size	Pop dataset				Pooled dataset		
	Frame 0	Frame 1	Frame 2		Frame 0	Frame 1	Frame 2
**20**	0/6	ND*	9/0		6/15	6/0	9/0
**21**	15/6	9/15	9/0		15/6	9/0	9/18
**22**	9/0	9/18	18/9		9/15	9/0	18/9
**23**	9/15	12/15	18/12		9/15	9/18	18/12
**24**	15/12	12/18	18/12		15/9	12/15	18/12
**25**	12/15	12/18	18/12		15/12	12/15	18/12
**26**	15 /12	15/18	18/15		15 /12	12/15	18/15
**27**	**15**	15/18	18/15		**15**	**15**	18/15
**28**	**15**	18/15	**18**		**15**	**15**	18/15
**29**	**15**	18/15	**18**		**15**	15/18	**18**
**30**	**15**	**18**	**18**		**15**	18/15	**18**
**31**	18/15	**18**	**18**		**15**	**18**	**18**
**32**	18/15	**18**	**18**		18/15	**18**	**18**
**33**	18/0	18/0	ND		18/15	**18**	18/15
**34**	ND	ND	ND		18/15	18/15	18/21
**35**	ND	ND	ND		18/15	15/18	21/18

*ND = Not Defined. The number of genes for certain S and F combinations are less than 10 and hence Integer Programming algorithm is not applied to these combinations.

Supplementary Table S3: For unique offsets described in Table 1, the robustness to variation in parameters and consistency across different Ribo-Seq datasets are described with additional sub columns. The two sub columns in the top row refers to the unique offsets being sensitive (S) or robust (R) to parameter variation. Namely, the change in threshold from 60% to 80% to classify the most probable offset as unique (left sub column) and variation in threshold of the secondary selection criterion $R(1) < a * Mean\big(R(2), R(3), R(4)\big)$ where a ranges from 1 to $\frac{1}{10}$ (right sub column). The bottom row specifies the consistency of the unique offset across individual Ribo-Seq datasets. For example, for fragment size 27 in frame 0, 15 is the unique offset which is sensitive (S in left sub column) to a change in threshold from 60% to 80% and robust (R in right sub column) to change in secondary selection parameter a from 1 to $\frac{1}{10}$. It is also consistent in 9 out of 12 datasets for which we have more than 10 genes meeting our filtering criteria.

Fragment Size	Frame 0				Frame 1				Frame 2			
**24**	**15**	R	R	4 of 4	15/12				18/12			
**25**	**15**	S	R	5 of 7	12/15				**18**	S	S	4 of 4
**26**	15/12				18/15				18/15			
**27**	**15**	S	R	9 of 12	**15**	S	R	7 of 12	**18**	R	R	6 of 9
**28**	**15**	R	R	14 of 17	**15**	S	R	10 of 13	**18**	R	R	10 of 12
**29**	**15**	R	R	14 of 15	15/18				**18**	R	R	15 of 16
**30**	**15**	R	R	15 of 15	**18**	R	R	12 of 16	**18**	R	R	16 of 16
**31**	**15**	S	R	12 of 13	**18**	R	R	13 of 15	**18**	R	R	15 of 16
**32**	18/15				**18**	R	R	11 of 11	**18**	R	R	9 of 10
**33**	**18**	S	S	6 of 6	**18**	R	R	7 of 7	**18**	S	S	4 of 4
**34**	**18**	R	R	2 of 2	**18**	R	S	2 of 2	18/21			

Supplementary Table S4: Input A-site offset tables used in the creation of artificial Ribo-Seq data (Top row, see Methods). Offset A-site tables (Bottom row) output by the Integer Programming method when applied to artificial Ribo-Seq data constructed using the input tables (Top) and P(S, F) distribution with mode $(28, 0)$ and variance $\lambda = 48$ (Distribution 5 in Fig S3).

Input Offset tables

Fragment size	Constant offset of 15			Constant offset of 18			Mixed offsets of 12 and 18			Top offsets from exp. data		
	Frame 0	Frame 1	Frame 2	Frame 0	Frame 1	Frame 2	Frame 0	Frame 1	Frame 2	Frame 0	Frame 1	Frame 2
20	15	15	15	18	18	18	12	12	12	6	6	9
21	15	15	15	18	18	18	12	12	12	15	9	9
22	15	15	15	18	18	18	12	12	12	15	6	18
23	15	15	15	18	18	18	12	12	12	15	18	18
24	15	15	15	18	18	18	12	12	12	15	15	12
25	15	15	15	18	18	18	12	12	12	15	12	18
26	15	15	15	18	18	18	12	12	12	15	15	18
27	15	15	15	18	18	18	12	12	12	15	15	18
28	15	15	15	18	18	18	18	18	18	15	15	18
29	15	15	15	18	18	18	18	18	18	15	15	18
30	15	15	15	18	18	18	18	18	18	15	18	18
31	15	15	15	18	18	18	18	18	18	15	18	18
32	15	15	15	18	18	18	18	18	18	15	18	18
33	15	15	15	18	18	18	18	18	18	18	18	18
34	15	15	15	18	18	18	18	18	18	18	18	18
35	15	15	15	18	18	18	18	18	18	18	18	18

Output Offset tables

Fragment size	Constant offset of 15			Constant offset of 18			Mixed offsets of 12 and 18			Top offsets from exp. data		
	Frame 0	Frame 1	Frame 2	Frame 0	Frame 1	Frame 2	Frame 0	Frame 1	Frame 2	Frame 0	Frame 1	Frame 2
20	15	15	15	18	18	18	12	12	12	6	6	9
21	15	15	15	18	18	18	12	12	12	15	9	9
22	15	15	15	18	18	18	12	12	12	15	6	18
23	15	15	15	18	18	18	12	12	12	15	18	18
24	15	15	15	18	18	18	12	12	12	15	15	12
25	15	15	15	18	18	18	12	12	12	15	12	18
26	15	15	15	18	18	18	12	12	12	15	15	18
27	15	15	15	18	18	18	12	12	12	15	15	18
28	15	15	15	18	18	18	18	18	18	15	15	18
29	15	15	15	18	18	18	18	18	18	15	15	18
30	15	15	15	18	18	18	18	18	18	15	18	18
31	15	15	15	18	18	18	18	18	18	15	18	18
32	15	15	15	18	18	18	18	18	18	15	18	18
33	15	15	15	18	18	18	18	18	18	18	18	18
34	15	15	15	18	18	18	18	18	18	18	18	18
35	15	15	15	18	18/15	18/15	18	18	18	18	18	18/15

Supplementary Table S5: Initial offset table after application of Integer Programming algorithm to a Pooled dataset in mESCs consisting of all genes. Offset table after application of Integer Programming algorithm to a Pooled dataset of *E. coli.*

Fragment size	mESCs Pooled dataset				*E. coli* Pooled dataset		
	Frame 0	Frame 1	Frame 2		Frame 0	Frame 1	Frame 2
**20**	6/3	6/9	0/15		12/9	15/0	9/0
**21**	6/0	6/0	0/18		12/9	3/12	9/0
**22**	0/9	0/3	9/15		12/0	12/0	9/0
**23**	6/15	9/0	18/9		12/3	0/12	9/0
**24**	9/15	3/0	18/0		12/3	12/0	0/9
**25**	9/6	9/6	12/15		12/3	12/3	9/6
**26**	12/6	9/12	15/0		12/3	3/0	9/3
**27**	12/9	12/21	15/9		3/12	3/6	9/3
**28**	12/15	12/21	15/12		3/12	3/6	3/0
**29**	15/12	15/12	18/15		3/12	3/6	3/9
**30**	**15**	15/18	18/15		12/9	9/3	9/6
**31**	15/12	15/18	18/15		12/3	9/6	9/3
**32**	12/15	18/15	**18**		12/9	3/9	9/3
**33**	15/12	18/15	**18**		12/9	9/3	9/3
**34**	15/18	15/18	18/12		12/9	9/12	9/6
**35**	ND*	ND	ND		12/9	12/9	9/6
**36**	ND	ND	ND		12/15	9/12	9/12
**37**	ND	ND	ND		12/6	12/9	9/12
**38**	ND	ND	ND		12/15	12/15	9/12
**39**	ND	ND	ND		12/15	12/3	9/3
**40**	ND	ND	ND		12/15	15/3	9/3

*ND = Not Defined. The number of genes for certain S and F combinations are less than 10 and hence Integer Programming algorithm is not applied to these combinations.

Supplementary Table S6: A-site locations (nucleotide offsets from 5′ end) determined by applying the Integer Programming algorithm to the Pooled dataset in mESCs are shown as a function of fragment size and frame. The dataset consists of only genes that have a single isoform and only one translation start site. The top two offset values are listed for those S and F combinations in which the A-site location could not be uniquely determined. The description of the sub columns is the same as Supplementary Table S3.

<table>
<tr><th>Fragment Size</th><th colspan="3">Frame 0</th><th colspan="3">Frame 1</th><th colspan="3">Frame 2</th></tr>
<tr><td rowspan="2">28</td><td colspan="3" rowspan="2">15/12</td><td colspan="3" rowspan="2">15/12</td><td rowspan="2">15</td><td>S</td><td>R</td></tr>
<tr><td colspan="2">1 of 2</td></tr>
<tr><td rowspan="2">29</td><td rowspan="2">15</td><td>R</td><td>R</td><td colspan="3" rowspan="2">15/18</td><td colspan="3" rowspan="2">15/18</td></tr>
<tr><td colspan="2">2 of 2</td></tr>
<tr><td rowspan="2">30</td><td rowspan="2">15</td><td>R</td><td>R</td><td colspan="3" rowspan="2">15/18</td><td colspan="3" rowspan="2">18/15</td></tr>
<tr><td colspan="2">2 of 2</td></tr>
<tr><td rowspan="2">31</td><td rowspan="2">15</td><td>R</td><td>R</td><td rowspan="2">15</td><td>S</td><td>S</td><td rowspan="2">18</td><td>R</td><td>R</td></tr>
<tr><td colspan="2">2 of 2</td><td colspan="2">1 of 2</td><td colspan="2">2 of 2</td></tr>
<tr><td rowspan="2">32</td><td rowspan="2">15</td><td>R</td><td>R</td><td colspan="3" rowspan="2">18/15</td><td rowspan="2">18</td><td>R</td><td>R</td></tr>
<tr><td colspan="2">2 of 2</td><td colspan="2">2 of 2</td></tr>
<tr><td rowspan="2">33</td><td rowspan="2">15</td><td>R</td><td>R</td><td rowspan="2">18</td><td>R</td><td>R</td><td rowspan="2">18</td><td>R</td><td>R</td></tr>
<tr><td colspan="2">2 of 2</td><td colspan="2">2 of 2</td><td colspan="2">2 of 2</td></tr>
<tr><td rowspan="2">34</td><td colspan="3" rowspan="2">15/12</td><td rowspan="2">18</td><td>S</td><td>S</td><td rowspan="2">18</td><td>R</td><td>R</td></tr>
<tr><td colspan="2">2 of 2</td><td colspan="2">2 of 2</td></tr>
</table>

Supplementary Table S7: Number of genes in the combination of fragment size and frame meeting the criteria of at least 1 read per codon on average in mESCs and *E. coli* Pooled datasets. The mESCs Pooled dataset consist of genes that are single isoform and have only one defined translation initiation site.

Fragment size	mESCs Pooled dataset				*E. coli* Pooled dataset		
	Frame 0	Frame 1	Frame 2		Frame 0	Frame 1	Frame 2
**20**	8	7	10		313	243	330
**21**	10	0	1		440	270	377
**22**	10	1	10		532	416	431
**23**	15	15	18		610	471	645
**24**	41	20	19		806	471	655
**25**	61	19	52		816	610	603
**26**	52	43	75		765	625	742
**27**	73	38	45		952	681	825
**28**	126	55	119		1001	849	868
**29**	187	125	208		988	840	981
**30**	230	191	257		1072	791	956
**31**	197	192	280		1042	898	916
**32**	103	187	237		994	823	993
**33**	47	125	108		1060	761	943
**34**	17	55	45		1008	842	856
**35**	0	0	0		891	740	924
**36**	0	0	0		943	598	799
**37**	0	0	0		827	618	625
**38**	0	0	0		640	494	596
**39**	0	0	0		588	323	461
**40**	0	0	0		440	288	278

Supplementary Table S8: Median normalized ribosome densities for 61 codon types were correlated with tRNA abundance for the Integer Programming method and 11 other contemporary methods (see Methods for details). The tRNA abundance values were obtained from Table S2 of study of Weinberg and co-workers[4].

**Method**	**Spearman's rho**	**p-value**
Integer Programming	-0.583	6.39×10^{-5}
Heuristic +18	-0.581	6.76×10^{-5}
Plastid	-0.580	6.98×10^{-5}
RiboProfiling	-0.575	8.55×10^{-5}
riboWaltz	-0.574	8.65×10^{-5}
Hussmann	-0.571	9.53×10^{-5}
Martens	-0.571	9.82×10^{-5}
Heuristic +15	-0.570	9.94×10^{-5}
ribodeblur	-0.570	9.94×10^{-5}
Scikit-ribo	-0.567	1.09×10^{-4}
Rpbp	-0.566	1.12×10^{-4}
Center-weighted	-0.517	5.31×10^{-4}

Supplementary Table S9: Publicly available datasets used in the study.

Dataset (first author name)	Year of publication	Number of replicates*	GEO Study	Accession numbers of samples used
Saccharomyces cerevisiae				
Pop	2014	1	GSE63789	GSM1557447
Guydosh	2014	1	GSE52968	GSM1279568
Jan	2014	1	GSE61012	GSM1495525
Williams	2014	1	GSE61011	GSM1495503
Gerashchenko	2014	1	GSE59573	GSM1439584
Gardin	2014	2	GSE51164	GSM1239959, GSM1239960
Lareau	2014	3	GSE58321	GSM1406453, GSM1406454, GSM1406455
Nedialkova	2015	3	GSE67387	GSM1646015, GSM1646016, GSM1646017
Young	2015	1	GSE69414	GSM1700885
Weinberg	2016	1	GSE53268	GSM1289257
Nissley	2016	2	GSE75322	GSM1949550, GSM1949551
Mouse embryonic stem cells (mESCs)				
Ingolia	2011	1	GSE30839	GSM765298
Hurt	2013	1	GSE41785	GSM1024298
Escherichia coli				
Li	2012	2	GSE35641	GSM872393, GSM872394
Li	2014	1	GSE53767	GSM1300279
Woolstenhulme	2015	2	GSE64488	GSM1572273, GSM1572275

* For datasets with more than one replicate, all replicates were used to create the Pooled dataset.

Supplementary Table S10: A-site offsets determined using the publicly available R packages – Plastid[5] , RiboProfiling[6] and riboWaltz[7]. These methods generate a P-site offset as output for each fragment length. The A-site offsets below are obtained after adding 3 nt to the P-site offsets.

	***S. cerevisiae* Pop data**				***S. cerevisiae* Pooled data**				**mESCs Pooled data**		
Fragment size	Plastid	RiboProfiling	riboWaltz		Plastid	RiboProfiling	riboWaltz		Plastid	RiboProfiling	riboWaltz
20	16	7	16		16	7	16		NA	NA	NA
21	16	7	16		16	7	13		NA	NA	NA
22	16	7	15		16	7	15		NA	NA	NA
23	16	10	15		16	10	15		NA	NA	NA
24	16	10	17		16	10	15		NA	NA	NA
25	16	11	15		16	11	15		16	13	15
26	16	11	16		16	11	16		16	14	16
27	16	14	14		16	14	14		16	15	15
28	16	15	15		16	15	15		13	16	15
29	16	16	16		16	16	16		13	6	15
30	16	16	16		16	16	16		15	15	15
31	16	16	16		16	16	16		13	13	16
32	16	17	17		16	17	17		16	13	16
33	16	17	17		16	17	17		16	14	16
34	16	13	15		16	13	15		17	13	14
35	16	13	16		16	13	15		NA	NA	NA

SUPPLEMENTARY REFERENCES

1. Hazewinkel, M. *Encyclopedia of Mathematics*. (Springer Science+Business Media B.V. / Kluwer Academic Publishers).

2. Woolstenhulme, C. J., Guydosh, N. R., Green, R. & Buskirk, A. R. High-Precision analysis of translational pausing by ribosome profiling in bacteria lacking EFP. *Cell Rep.* **11,** 13–21 (2015).

3. Good, P. *Permutation, Parametric, and Bootstrap Tests of Hypothesis*. (Springer Series in Statistics, 2005). doi:10.1007/978-0-387-98135-2

4. Weinberg, D. E., Shah, P., Eichhorn, S. W., Hussmann, J. A., Plotkin, J. B. & Bartel, D. P. Improved Ribosome-Footprint and mRNA Measurements Provide Insights into Dynamics and Regulation of Yeast Translation. *Cell Rep.* **14,** 1787–1799 (2016).

5. Dunn, J. G. & Weissman, J. S. Plastid: nucleotide-resolution analysis of next-generation sequencing and genomics data. *BMC Genomics* **17,** 958 (2016).

6. Popa, A., Lebrigand, K., Paquet, A., Nottet, N., Robbe-Sermesant, K., Waldmann, R. & Barbry, P. RiboProfiling: a Bioconductor package for standard Ribo-seq pipeline processing. *F1000Research* **5,** 1309 (2016).

7. Lauria, F., Tebaldi, T., Bernabò, P., Groen, E. J. N., Gillingwater, T. H. & Viero, G. riboWaltz: Optimization of ribosome P-site positioning in ribosome profiling data. *PLoS Comput. Biol.* **14,** 1–20 (2018).
